# Differential role of chronic liver diseases on the incidence of cancer: a longitudinal analysis among 248,224 outpatients in Germany

**DOI:** 10.1007/s00432-022-04198-5

**Published:** 2022-07-22

**Authors:** Sven H. Loosen, David Schöler, Mark Luedde, Johannes Eschrich, Tom Luedde, Karel Kostev, Christoph Roderburg

**Affiliations:** 1 Clinic for Gastroenterology, Hepatology and Infectious Diseases, University Hospital Düsseldorf, Medical Faculty of Heinrich Heine University Düsseldorf, Moorenstrasse 5, 40225 Düsseldorf, Germany; 2 KGP Bremerhaven, Bremerhaven, Germany; 3 Clinic for Hepatology and Gastroenterology, Charité University Medical Center, Augustenbuger Platz 1, 13353 Berlin, Germany; 4 Epidemiology, IQVIA, Frankfurt, Germany

**Keywords:** HCC, Hepatocellular carcinoma, Fib-4, Biomarker, Score

## Abstract

**Background:**

Chronic liver diseases, especially chronic hepatitis, are a known risk factor for the development of liver cancer. However, the risk of total cancer development and malignant potential from these diseases is largely unknown. Systematic data on the risk of cancer development from these diseases are missing. Therefore, the goal of this study is to analyze the risk of total cancer development in chronic liver diseases.

**Methods:**

A cohort of 15,706 patients with chronic hepatitis and 15,706 patients without hepatitis were matched by propensity scoring from outpatient practices in Germany over a period of 15 years. Cox regression models were conducted to study the association between alcoholic hepatitis, autoimmune hepatitis, hepatitis B, hepatitis C and cancer incidence, including liver, other digestive organs, skin, prostate, breast and lymphoid and hematopoietic tissue cancer.

**Results:**

Within 10 years of the index date, 19.3% of patients with alcoholic hepatitis and 13.4% of non-hepatitis individuals were diagnosed with cancer (log-rank *p* = 0.035). These proportions were 15.0 vs. 9.9% (*p* = 0.078) for autoimmune hepatitis, 8.7 vs. 7.1% (*p* = 0.015) for hepatitis B, and 12.7 vs. 7.6% (*p* < 0.001) for hepatitis C. In regression analyses, only alcoholic hepatitis (HR: 1.84, 95% CI 1.32–2.54) and hepatitis C (HR: 2.10, 95% CI 1.77–2.50) were significantly associated with increased risk of cancer. There was a very strong positive association between hepatitis C and liver cancer (HR: 78.2 (95% CI 10.9–560.7). Furthermore, hepatitis C was associated with an increased risk of respiratory organ cancer (HR: 2.59, 95% CI 1.42–4.73).

**Conclusion:**

This study confirms the strong association between chronic hepatitis and liver cancer, but also with an overall elevated cancer risk, and especially of cancer in the respiratory tract in patients with chronic hepatitis C.

## Introduction

Chronic hepatitis is a known risk factor for the development of liver cancer. While chronic inflammation is a major driver in hepatocellular carcinoma (HCC) initiation and progression, hepatitis viruses can also directly induce HCC, e.g., through hepatitis B virus (HBV) DNA integration into the host cell genome (Tu et al. 2017). Chronic infections due to hepatitis B and hepatitis C are responsible for most cases of HCC worldwide (Mattos et al. 2021; Tsukuma et al. 1993), and despite available vaccination against hepatitis B and effective treatments against hepatitis C, there were still 296 million patients with chronic hepatitis B and 58 million patients with chronic hepatitis C worldwide in 2019, according to the WHO (Global progress report on HIV, viral hepatitis and sexually transmitted infections 2021).

Recently, it has been shown by a systematic review that hepatitis C is associated with an increased risk of lung cancer (Ponvilawan et al. 2020). In addition, cancer rates for other malignancies have been shown to be higher in hepatitis C, e.g., esophageal, pancreas, renal, stomach, or colorectal cancer (Nyberg et al. 2020). Another meta-analysis of 17 studies found that HBV increases the risk of pancreatic cancer (Liu et al. 2021). In addition, HBV/HDV co-infected patients are known to be at a twofold increased risk of HCC compared to HBV mono-infected patients (Kamal et al. 2021; Puigvehí et al. 2019). Another Swedish study found an increased risk for any cancer in patients with autoimmune hepatitis (Sharma et al. 2022) and in patients with alcoholic liver disease, an increased risk of gastric cancer was found (Ha et al. 2017). Besides the elevated risk of liver cancer, it is also known that there is an increased risk for extra-hepatic malignancies in patients with cirrhosis, although several confounding factors, such as tobacco consumption or alcohol intake have to be considered (Sørensen et al. 1998).

Systematic analyses on the overall cancer rate are missing, and it is currently unclear to what extent the development of several malignancies is increased in different forms of liver diseases. We, therefore, used a large patient database to analyse the association between alcoholic hepatitis, autoimmune hepatitis, hepatitis B, hepatitis C and cancer incidence and subsequent cancer diagnosis.

## Materials and methods

### Database

This study was based on data from the Disease Analyzer database (IQVIA), which contains drug prescriptions, diagnoses, and basic medical and demographic data obtained directly and in anonymous format from computer systems used in the practices of general practitioners and specialists (Rathmann et al. 2018). The database covers approximately 3% of all outpatient practices in Germany. Diagnoses (according to International Classification of Diseases, 10th revision [ICD-10]), prescriptions (according to Anatomical Therapeutic Chemical [ATC] Classification system), and the quality of reported data are being monitored by IQVIA. In Germany, the sampling methods used to select physicians' practices are appropriate for obtaining a representative database of general and specialized practices. It has previously been shown that the panel of practices included in the Disease Analyzer database is representative of general and specialized practices in Germany (Rathmann et al. 2018). Finally, this database has already been used in previous studies focusing on cancer (Loosen et al. 2022; Jacob et al. 2022).

### Study population

**Fig. 1:**
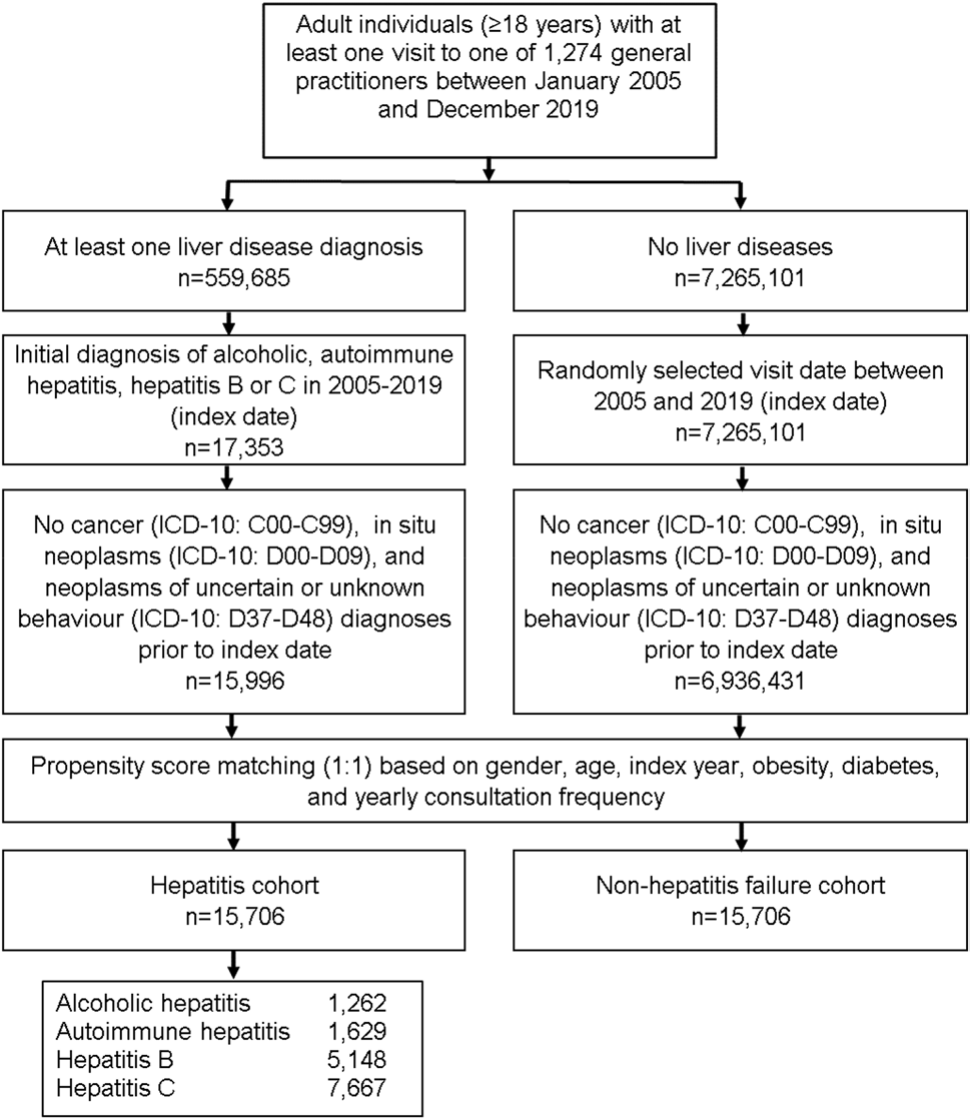
Selection of study patients

This retrospective cohort study included adult patients (≥ 18 years) with an initial diagnosis of chronic hepatitis including alcoholic hepatitis (ICD-10: K70.1), autoimmune hepatitis (ICD-10: K75.4), hepatitis B (ICD-10: B18.0, B18.1) and hepatitis C (ICD-10: B18.2) in 1274 general practices in Germany between January 2005 and December 2019 (index date; Fig. 1). Patients with cancer diagnoses (ICD-10: C00-C99), in situ neoplasms (ICD-10: D00-D09), and neoplasms of uncertain or unknown behavior (ICD-10: D37-D48) prior to or at index date were excluded. Hepatitis patients were matched to individuals without hepatitis by propensity scores based on sex, age, index year, obesity, diabetes, and yearly consultation frequency. Diabetes and obesity were used as they are associated with cancer. As hepatitis patients have much higher consultation frequency by GPs, and higher consultation frequency can increase the probability of other diagnoses documentation, we included consultation frequency per year in the matching. For the non-hepatitis cohort, the index date was that of a randomly selected visit between January 2005 and December 2019 (Fig. 1).

### Study outcomes and covariates

The main outcome of the study was the incidence of cancer (ICD 10: C00-C97) in total and cancer of different organs including digestive organs excluding liver (ICD 10: C15-C26 excl. C22), liver (ICD-10: C22), respiratory organs (ICD 10: C30-C39), skin (ICD 10: C43, C44), breast (ICD 10: C50), prostate (ICD 10: C61), and lymphoid and hematopoietic tissue (ICD 10: C81-C96) as a function of chronic hepatitis.

### Statistical analyses

Differences in the sample characteristics between those with and those without chronic hepatitis were tested using chi-squared tests for categorical variables and Wilcoxon tests for continuous variables. Cumulative incidence of cancer was evaluated using Kaplan–Meier curves. Cox regression models were conducted to study the association between alcoholic hepatitis, autoimmune hepatitis, hepatitis B, hepatitis C and cancer incidence. Each kind of hepatis was compared with accordingly matched non-hepatitis individuals. To counteract the problem of multiple comparisons, *p* values < 0.001 were considered statistically significant. Analyses were carried out using SAS version 9.4 (SAS institute, Cary, NC, USA).

## Results

### Basic characteristics of the study sample

The present study included a total of 15,706 patients with chronic hepatitis as well as 15,706 matched individuals without chronic hepatitis. The basic characteristics of the study cohort are shown in Table 1. The mean age [SD] was 48.4 [19.2] years and 44.1% of patients were female. The average follow-up period for all patients was 4.1 (SD: 3.9) years. The chronic hepatitis cohort and the non-hepatitis cohort had an average follow-up time of 4.7 (SD: 4.3) and 3.3 (SD: 3.1) years, respectively. During the follow-up period, the mean annual number of GP visits was 2.2 (SD: 5.1) times. Of all 15,706 patients with chronic hepatitis, 1,262 patients had a diagnosis of alcoholic hepatitis (mean age 53.4: (SD: 12.5) years) and 1,629 patients were diagnosed with autoimmune hepatitis (mean age: 55.9 (SD: 17.0) years). 5,148 and 7667 patients had a diagnosed hepatitis B (mean age: 47.7 (SD: 15.4) years) and hepatitis C (mean age: 48.6 (SD: 14.9) years), respectively.

**Table 1:** Basic characteristics of the study sample (after 1:1 matching)

Variable	Proportion affected among patients with chronic hepatitis (%) *N* = 15,706	Proportion affected among patients without chronic hepatitis (%) *N* = 15,706	*p* value
Age (Mean, SD)	48.4 (19.2)	48.4 (19.2)	1.000
Age ≤ 40	32.9	32.9	1.000
Age 41–50	25.5	25.5	
Age 51–60	21.1	21.1	
Age > 60	20.6	20.6	
Women	44.1	44.1	1.000
Men	55.9	55.9	
Diabetes	11.3	11.3	1.000
Obesity	8.0	8.0	1.000
Yearly consultation frequency	2.2 (5.1)	2.2 (5.1)	1.000

Proportions of patients given in %, unless otherwise indicated

*SD* standard deviation

## Association between chronic hepatitis and the incidence of cancer

**Fig. 2:**
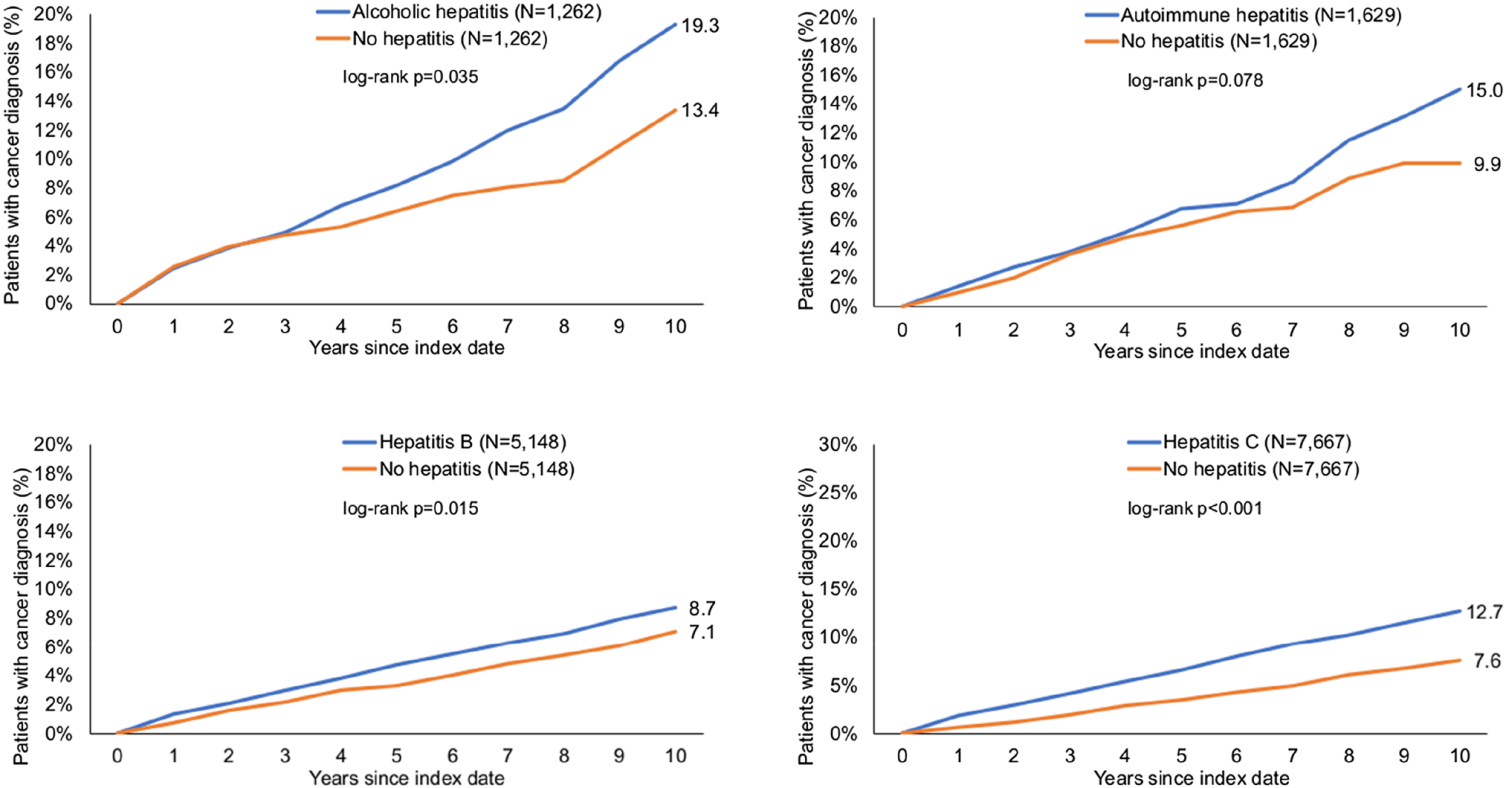
Kaplan–Meier curves for time to cancer diagnosis in patients with and without chronic hepatitis

First, we investigated a possible association between chronic hepatitis of different causes and the occurrence of cancer. Within 10 years from the index date, 19.3% of patients with alcoholic hepatitis and 13.4% of non-hepatitis individuals were diagnosed with cancer (log-rank *p* = 0.035). These proportions were 15.0 vs. 9.9% (*p* = 0.078) for autoimmune hepatitis. In addition, both hepatitis B (8.7 vs. 7.1%, *p* = 0.015) and hepatitis C (12.7 vs. 7.6%, *p* < 0.001) in particular significantly increased the proportion of patients diagnosed with cancer during the follow-up period (Fig. 2). The overall incidence rate of cancer for the cohort of patients with alcohol hepatitis, autoimmune hepatitis, hepatitis B, hepatitis C and individuals without hepatitis were 20.6, 15.5, 9.7, 14.2 and 5.7 cases per 1,000 patient years, respectively (Table 2).

**Table 2:** Incidence of cancer cases per 1000 patient years

Cohort	Observation time in patient years	Cancer cases	Incidence in cases per 1000 patient years
Alcoholic hepatitis	6415	132	20,6
Autoimmune hepatitis	7614	118	15,5
Hepatitis B	24,460	237	9,7
Hepatitis C	32,876	466	14,2
No hepatitis	71,366	410	5,7

## Association between chronic hepatitis and different subtypes of cancer

To further dissect the association between chronic liver disease and the development of cancer, we subsequently evaluated the influence of the different etiologies of chronic hepatitis on cancer development with respect to different tumor sites. In regression analyses, only alcoholic hepatitis (HR: 1.84, 95% CI 1.32–2.54) and hepatitis C (HR: 2.10, 95% CI 1.77–2.50) were significantly associated with an increased risk of cancer development in general. When looking at individual tumor sites, we observed a very strong and significant positive association between hepatitis C and the development of liver cancer (HR: 78.2, 95% CI 10.9–560.7, Table 3). HRs regarding the development of liver cancer could not be calculated for the other underlying etiologies, as there were no diagnoses of liver cancer among the matched non-hepatitis individuals in these groups (Table 3). Finally, we observed a positive association between chronic hepatitis due to hepatitis C infection and an increased risk of respiratory organ cancer (HR: 2.59, 95% CI 1.42–4.73, Table 3). In addition, we observed a trend towards a positive correlation between hepatitis C and the development of skin cancer (HR: 1.55, 95% CI 1.01–2.83) as well as digestive organ cancer excl. liver (HR: 1.70, 95% CI 1.11–2.59, Table 2) but the adjusted level of statistical significance was not reached. There were no significant correlations between the other disease etiologies and individual cancer sites including prostate cancer, breast cancer and cancer of the lymphoid and haematopoietic tissue (Table 3).

**Table 3:** Association between chronic hepatitis and the incident cancer diagnoses in patients followed in general practices in Germany (Cox regression models)

Cancer site	Alcoholic hepatitis vs. no hepatitis	Autoimmune hepatitis vs. no hepatitis	Hepatitis B vs. no hepatitis	Hepatitis C vs no hepatitis
Cancer total	1.84 (1.32–2.54)*	1.29 (0.95–1.76)	1.25 (1.00–1.55)	2.10 (1.77–2.50)*
Liver	–^a^	–^b^	–^c^	78.17 (10.90–560.69)*
Digestive organs excl. liver	1.83 (0.80–4.16)	1.70 (0.71–4.08)	1.93 (0.94–3.94)	1.70 (1.11–2.59)
Respiratory organs	1.61 (0.70–3.89)	1.38 (0.34–5.55)	0.77 (0.37–1.62)	2.59 (1.42–4.73)*
Skin	1.82 (0.76–4.36)	1.07 (0.57–2.00)	0.84 (0.47–1.50)	1.55 (1.01–2.83)
Prostate (men)	0.84 (0.34–2.04)	0.83 (0.25–2.74)	0.67 (0.29–1.52)	0.94 (0.48–1.82)
Breast (women)	2.22 (0.45–11.01)	1.57 (0.68–3.63)	0.86 (0.44–1.68)	1.04 (0.60–1.81)
Lymphoid and haematopoietic tissue	1.72 (0.66–4.44)	1.83 (0.72–4.66)	1.39 (0.77–2.51)	1.79 (1.14–2.83)

*p < 0.01

^a^9 cases among alcoholic hepatitis cohort and 0 cases among non-hepatitis cohort

^b^4 cases among autoimmune hepatitis cohort and 0 cases among non-hepatitis cohort

^c^35 cases among hepatitis B cohort and 0 cases among non-hepatitis cohort

## Discussion

Chronic liver diseases are a well-established risk factor for the development of liver cancer, while their role in total cancer development is much less clear since systematic data on the risk of cancer development from these diseases are scarce. We, therefore, analyzed an association between chronic hepatitis and subsequent cancer diagnosis in a cohort of 15,706 patients with chronic hepatitis and 15,706 matched patients without hepatitis. Notably, our data confirmed the strong relationship of chronic hepatitis not only with liver cancer, but also with an overall elevated cancer risk, especially that of respiratory tract cancers in patients with chronic hepatitis C.

We demonstrate an association with a numerically elevated risk for all sites cancer in patients with chronic hepatitis of all etiologies. In regression analyses, only alcoholic hepatitis (HR: 1.84, 95% CI 1.32–2.54) and hepatitis C (HR: 2.10, 95% CI 1.77–2.50) were significantly associated with an increased risk of cancer. While we found a striking (but expected) increase of liver cancer risk in patients with hepatitis C, cases of liver cancer were too rarely in the other etiologies of chronic hepatitis to show any statistical association. Nevertheless, it is important to note that 9 cases of liver cancers were found among the alcoholic hepatitis cohort, 4 cases among the autoimmune hepatitis cohort and 35 cases among the hepatitis B cohort, while no case was found in the respective matched controls. These data clearly show the relevance of chronic hepatitis as a liver cancer risk factor independent of the specific disease etiology and underline the value of our analyses by confirming this well-established association. Along with many previous analyses, our data clearly confirm the need for prevention and screening examinations in this high-risk cohort to ensure the earliest possible detection of liver tumors. Such an early detection allows in many cases to identify patients in curatively treatable stages of the disease and thus to enable long-term survival.

While the role of chronic hepatitis as a risk factor for liver cancer is well established and prototypic for the association between an inflammatory disease and the development of malignant disease, the relationship between chronic liver disease and other cancers is far less well understood. In our cohort, we found significantly higher rates of respiratory tract cancers in patients with hepatitis C virus infection. This important finding of our analysis is in line with a recent analysis reporting an elevated lung cancer risk (1.6 [1.3–1.9]) in 12,126 chronic HCV-infected persons in the Chronic Hepatitis Cohort Study (CHeCS) (Allison et al. 2015). However, we are unable to clearly state on the background for these observations. It seems likely that common risk factors for hepatitis C virus infections and respiratory tract cancers rather than a direct causal relationship between both diseases might explain higher rates of respiratory tract cancer in hepatitis C virus patients. As such, hepatitis C virus infections are more common in patients with a lower socioeconomic level, in which higher rates of tobacco abuse as an important risk factor for respiratory tract cancers are found as it was reported for the Chronic Hepatitis Cohort Study (Allison et al. 2015). Because we were unable to control for tobacco use, it remains unclear whether this difference in smoking rates could account for the differences in incidence rates. This and other confounders were not included in this study, which is a major limitation. These factors will have to be included in future investigations to control for confounding effects. Interestingly, a similar association between chronic hepatitis and respiratory tract cancer was not found in our cohort of patients with alcoholic hepatitis, potentially arguing for a specificity of the observation. Thus, the possibility that the hepatitis C virus may be directly involved in the carcinogenesis of organs other than liver cannot be fully excluded. In a recent systematic review of the literature, Fiorini et al. (2015) suggested an increased prevalence of B-cell Non-Hodgkin-Lymphoma, intra-hepatic cholangiocarcinoma and pancreatic cancer in patients with HCV but concluded that due to the limited amount of data, no final conclusion regarding the relationship between HCV and extra-hepatic cancers can be made. In addition to these cancers, we found an association between skin cancers and presence of hepatitis C. In line with our data, higher rates of non-epithelial skin cancers were observed in a registry-based case–control study using the SEER-Medicare data in 1,623,538 US adults aged ≥ 66 years (adjusted OR 1.78 (1.31–2.43), *p* = 0.0002; (Mahale et al. 2017)). Similar to respiratory tract cancers, associations between HCV with non-epithelial skin cancers may be explained by confounding by shared risk factors. Here, also a detailed analysis of confounding factors is necessary to unravel causal relationships in these diseases. As such, the risk of non-epithelial skin cancers, including Merkel cell carcinoma is increased in people with HIV infection (Lanoy et al. 2009).

We did not observe associations between the other etiologies of chronic hepatitis and specific cancer sites, which is most probably due to small sample size. Moreover, we did not detect significant associations between chronic hepatitis and digestive organs excl. liver, prostate, breast and lymphoid and hematopoietic tissue, which were partially detected in other studies (Allison et al. 2015; Su et al. 2012). Aside from too small sample sizes, variation across studies may be due to differences in the populations, or differences in methods of exposure and outcome ascertainment. Moreover, different study designs might have influenced outcome of different studies, potentially leading to inter-study differences. We acknowledge the fact that our study is subject to various limitations, most of which are due to the chosen study design and cannot be avoided. Most importantly, diagnoses within our database are coded as ICD-10 codes, which might be associated with the misclassification of certain diagnoses. Furthermore, data on the socioeconomic status (e.g., education and income of patients) as well as lifestyle-related risk factors (e.g., smoking, alcohol consumption, and physical activity) are lacking within the Disease Analyzer database and thus cannot be taken into account in our study, but will be crucial for valid analysis of causal relationships. However, the IQVIA Disease Analyzer database used for the present analyses has been used extensively for various academic publications (e.g., (Loosen et al. 2022; Jacob et al. 2022)) and its validity has been well demonstrated (Rathmann et al. 2018). Next, subgroup analyses of individual cancer sites (e.g., left/right sided colorectal cancer) were not feasible due to the lack of information of the sides. We, therefore, grouped different tumor entities with similar pathomechanisms (e.g., digestive or respiratory organs), which might be associated with a presentation bias as described in detail in a recent study (Loosen et al. 2021). Finally, the information regarding disease stage (e.g., liver function) was not available within our database leading to the over- or underestimation of effects.

In summary, by analyzing data from a large German primary care provider database, we demonstrate that the presence of chronic liver diseases is associated with an increased risk of cancer regardless of the disease etiology and irrespective of patients’ age and sex. Notably, the effect on cancer development was not restricted to liver cancers since patients with hepatitis C virus infections displayed higher rates of respiratory tract and liver cancer. Thus, along with previous data, our study suggests that the clinical management of these patients should include a careful and structured risk assessment for the development of cancer in order to improve long-term outcomes in these patients. For example, patients with hepatitis C virus infections might be presented in a specific “board” and discussed with dedicated infectiologists and oncologists allowing cancer detection at the earliest possible time point.

## Data Availability

The data that support the findings of this study are available on request from the corresponding author.
